# Aspergillus and Candida in primary and secondary immunodeficiencies in USIDNET

**DOI:** 10.64898/2026.09.11.26362658

**Published:** 2026-09-12

**Authors:** Samantha Y. Starkey, USIDNET Consortium, Kathleen E Sullivan

## Abstract

**Objective:**

Fungal infections can represent opportunistic infections leading to the diagnosis of immunocompromise. Specific inborn errors of immunity are associated with aspergillus but it is unclear how sensitive aspergillus is for the subsequent diagnosis of those inborn errors of immunity.

**Methods:**

The patient registry USIDNET was utilized to extract clinical conditions and laboratory values from patients with candida and aspergillus diagnosis codes. We restricted the diagnoses to deep infections and then identified the associated clinical conditions along with potential settings, such as bone marrow transplant.

**Results:**

We identified 41 patients with aspergillus as a diagnosis code. The types of diagnoses associated with aspergillus included T cell disorders, antibody disorders, as well as the anticipated myeloid disorders. Bone marrow transplant was identified as a potential predisposing condition. 64. Patients were identified with candida as a diagnosis code. Similarly, T cell disorders, antibody disorders, and myeloid disorders were found.

**Conclusions:**

Candida and aspergillus are generally considered sentinel infections for myeloid disorders and T cell disorders. However, we found these infections associated with a broad range of inborn errors of immunity. This suggests that diagnostic testing should be broadened when facing patients with these infections.

## Full Text Availability

The license terms selected by the author(s) for this preprint version do not permit archiving in PMC. The full text is available from the preprint server.

